# Characteristics of galacturonate reductase (GalUR) genes in garlic (Allium sativum L.) and changes in their expression in response to abiotic stressors

**DOI:** 10.18699/vjgb-26-45

**Published:** 2026-05

**Authors:** M.A. Filyushin, T.M. Seredin, A.V. Shchennikova, E.Z. Kochieva

**Affiliations:** Skryabin Institute of Bioengineering, Federal Research Centre “Fundamentals of Biotechnology” of the Russian Academy of Sciences, Moscow, Russia; Skryabin Institute of Bioengineering, Federal Research Centre “Fundamentals of Biotechnology” of the Russian Academy of Sciences, Moscow, Russia; Skryabin Institute of Bioengineering, Federal Research Centre “Fundamentals of Biotechnology” of the Russian Academy of Sciences, Moscow, Russia; Skryabin Institute of Bioengineering, Federal Research Centre “Fundamentals of Biotechnology” of the Russian Academy of Sciences, Moscow, Russia

**Keywords:** garlic, Allium sativum L., L-ascorbic acid biosynthesis, D-galacturonic pathway, D-galacturonate reductase, GalUR, чеснок, Allium sativum L., биосинтез L-аскорбиновой кислоты, D-галактуроновый путь, D-галактуронатредуктаза, GalUR

## Abstract

In plants, the synthesis of L-ascorbic acid (Aa), in addition to the main L-galactose pathway, is carried out by three known alternative pathways. One of them, the D-galacturonic acid pathway, is thought to be specific for tissues with excess D-galacturonate, the substrate of D-galacturonate reductase (GalUR), which belongs to the Aldo-Keto Reductase (AKR) superfamily. In this study, the AKR gene family of garlic Allium sativum L. was identified and seven genes, AsGalUR1–7, presumably encoding GalUR enzymes, were determined. The structure and phylogeny of the AsGalUR1–7 genes and the proteins they encode, as well as the AsGalUR1–7 expression pattern in different organs of the garlic plant (in silico and qRT-PCR), were characterized. Based on the obtained data, the genes were conditionally divided into root (AsGalUR1–4) and leaf (AsGalUR5–7) groups depending on the highest expression level in the underground and aboveground parts of the plant, respectively. The AsGalUR expression in leaves and roots was analyzed in response to drought, salt and cold stresses, as well as exogenous phytohormones (abscisic acid, methyl jasmonate), accompanied by the AsA content measurement. It was shown that hormone treatment suppresses the expression of all analyzed genes in both organ types. Cold conditions stimulate the expression of root group genes and suppress that of leaf group genes in roots, and have the opposite effect in leaves. Osmotic stressors (NaCl, PEG) suppress the transcription of all genes in leaves, but do not change (NaCl) or stimulate (PEG) it in roots, which is accompanied by an increase in AsA accumulation in organs of both types. A positive correlation between the expression of the AsGalUR1 and 4 genes and the AsA content is found in leaves under stress conditions. The data obtained can form the basis for further study of the mechanisms regulating AsA synthesis in garlic and other Allium species.

## Abstract (ru)

Характеристика генов галактуронатредуктаз (GalUR) чеснока (Allium sativum L.) и изменение их экспрессии в ответ на абиотические стрессоры

## Introduction

L-ascorbic acid (AsA, ascorbate) is a major component of the non-enzymatic antioxidant system in plants (Smirnoff and Wheeler, 2024). The required level of ascorbate in cells is maintained through a balance between the biosynthesis, degradation, and recycling (reduction of oxidized forms) of AsA, as well as ascorbate transport (Smirnoff, 2018). AsA is synthesized primarily in leaves (Franceschi, Tarlyn, 2002; Badejo et al., 2012) and transported via the phloem to various plant parts in the form of stable and oxidation-protected ascorbyl glycosides (Richardson et al., 2021; Huang et al., 2025).

In plants, AsA biosynthesis occurs primarily via the Smirnoff– Wheeler L-galactose pathway. However, there are three alternative pathways – D-galacturonic, L-gulose, and myoinositol (Broad et al., 2020; Smirnoff, Wheeler, 2024), which also significantly contribute to ascorbate accumulation in some organs or at certain stages of plant development. For example, in strawberry (Fragaria × ananassa Duchesne ex Rozier), the L-galactose pathway is characteristic of leaves, while the D-galacturonic pathway is characteristic of ripe berries (Agius et al., 2003; Cruz-Rus et al., 2011; Liu et al., 2022). Similarly, in tomato (Solanum lycopersicum L.), leaves and growing fruits synthesize AsA via the L-galactose pathway, whereas ripe fruits, via the D-galacturonic pathway (Badejo et al., 2012). The transition from the main AsA biosynthetic pathway to an alternative variant in ripe fruits is associated with the active degradation of cell wall pectin, formed mainly by galacturonic acid residues, and the conversion of its main monomer, D-galacturonate, to AsA (Peltonen, Richard, 2022).

Of the alternative pathways, the D-galacturonic acid pathway is the best studied. It begins, as mentioned above, with the breakdown of pectin, resulting in the formation of D-galacturonic acid, which is converted to D-galacturonate (D-GalUA), followed by reduction to L-galactonate catalyzed by D-galacturonate reductase. L-galactonate is converted by aldonolactonase to L-galactono-1,4-lactone, which is oxidized to AsA by L-galactono-1,4-lactone dehydrogenase (Ishikawa et al., 2008; Peltonen, Richard, 2022).

Galacturonate reductases (EC 1.1.1.365; GalUR) structurally belong to the Aldo-Keto-Reductase (AKR) superfamily (Duan et al., 2020), one of 16 subfamilies of which, AKR4, includes AKR proteins of plant origin (Penning, 2015). The number of AKR genes identified in higher plants (Sengupta et al., 2015) varies significantly depending on the species. For example, the genome of Arabidopsis thaliana L. contains 22 AKR genes, that of S. lycopersicum, 25 genes, and that of F. × ananassa, 80 genes (Duan et al., 2020; Liu et al., 2022), and the functions of their protein products are not limited to participation in the synthesis of AsA. Plant AKRs also control the formation of other secondary metabolites and osmolytes, including vitamin B6, sorbitol, isoflavones, phytoestrogens, and others (Sengupta et al., 2015; Ha et al., 2019). Such functional differentiation of AKR proteins is considered to enhance plant adaptation to various environmental conditions, including stress factors. Regarding the AsA synthesis, it should be noted that the AKR family includes not only the GalUR enzymes of the D-galacturonic pathway, but also L-galactose dehydrogenase (L-GalDH) of the main L-galactose pathway (Vargas et al., 2022).

Garlic (Allium sativum L.) is an economically significant vegetable crop with an annual production of approximately 30 million tons (FAO; http://www.fao.org). The ascorbate content in leaves and bulbs has been extensively studied in various garlic varieties and accessions (Skoczylas et al., 2023; Šnirc et al., 2023; Popa et al., 2024; Yenealem et al., 2025). However, currently available information on the molecular regulation of AsA metabolism in A. sativum and equally economically valuable related Allium species is limited to research data from our laboratory. Namely, in A. sativum, individual genes of monodehydroascorbate reductases (MDHAR) of the ascorbate recycling pathway were identified and characterized (Anisimova et al., 2022), and changes in the expression of AsA biosynthesis and recycling genes in response to infection of garlic plants with the pathogenic fungus Fusarium proliferatum were shown (Shchennikova et al., 2025). In leek (Allium porrum L.), the variability of the GDP-L-galactose phosphorylase gene GGP1 of the L-galactose pathway was determined (Anisimova et al., 2021a), and possible dependences of the AsA content on the expression level of the MDHAR genes (Anisimova et al., 2021b; Filyushin et al., 2021) and some other genes of the L-galactose and AsA recycling pathways (Filyushin et al., 2025) were identified.

The present study was aimed to identify and characterize D-galacturonate reductase (GalUR) genes in the genome of garlic A. sativum and to determine their expression pattern in various plant organs, as well as in response to abiotic stress factors and exogenous phytohormones.

## Materials and methods

Identification and structural characterization of garlic AsGalUR genes. The D-galacturonate reductase gene sequences were searched for in the genomic and transcriptomic data of garlic A. sativum cv. Ershuizao (PRJNA606385, assembly Garlic.V2.fa) available in the AlliumDB database (https://allium.qau.edu.cn/). The GalUR sequences of tomato (LOC101256763 and LOC101250974 (=SlAKR4B)) (Suekawa et al., 2016) and strawberry (FaGalUR; AF039182.1) (Agius et al., 2003) were used as reference.

Sequence alignment and analysis were performed using MEGA 7.0 (https://www.megasoftware.net/). For the AsGalUR proteins, conserved domains and motifs (NCBI-CDD, http://www.ncbi.nlm.nih.gov/Structure/cdd/wrpsb.cgi; MEME 5.5.7, http://meme-suite.org/tools/meme), molecular weight (Mw) and isoelectric point (pI) (ExPASy, https://web. expasy.org/protparam/), molecular function and cellular localization (in terms of Gene Ontology, GO, PANNZER, http:// ekhidna2.biocenter.helsinki.fi/sanspanz/) were determined. Phylogenetic analysis was performed using AKR sequences of A. sativum, S. lycopersicum and A. thaliana (MEGA 7.0, Neighbor-Joining method, bootstrap based on 1,000 replicates).

Analysis of AsGalUR gene expression in different garlic organs. The organ-specific expression pattern of the identified AsGalUR genes was determined using two methods: in silico and real-time PCR (qRT-PCR).

In silico analysis of AsGalUR gene expression was performed using available transcriptome data of different organs of the garlic cv. Ershuizao (roots, leaves, stems, seedlings, buds, flowers, and bulbs at developmental stages 1–8) (Sun et al., 2020). The data were visualized as a heatmap (Heatmapper, http://www.heatmapper.ca/expression/), where expression levels were presented as fragments per kilobase per million mapped reads (FPKM).

Using qRT-PCR, the expression of AsGalUR genes was determined in the roots, base, bulb, pseudostem, and leaves of garlic cv. Scorpion plants grown in soil under greenhouse conditions (ECCF, Federal Research Center of Biotechnology, Russian Academy of Sciences; 16-h photoperiod (light phase from 8:00); day/night – 22/16 °C; lighting intensity 190 μM/ m2/s). The plant material was ground in liquid nitrogen and used for the isolation of total RNA with purification from DNA impurities (RNeasy Plant Mini Kit and RNase-free DNasy set; QIAGEN, Germany) and cDNA synthesis (GoScript™ Reverse Transcription System, Promega, USA).

Based on the identified AsGalUR sequences, specific primers for qRT-PCR were developed (Table 1). GAPDH (MZ171220.1) and UBQ (MZ171222.1) were used as reference genes. The reaction mixture included 3 ng of cDNA and the “Reaction mixture for qRT-PCR in the presence of SYBR Green I and ROX” kit (Synthol LLC, Russia). The reaction was carried out in a CFX96 Real-Time PCR Detection System (Bio-Rad Laboratories, USA), in six technical replicates of two biological replicates under the following conditions: 95 °C for 5 min; 40 cycles (95 °C for 15 s, 62 °C for 50 s). Data were statistically processed using one-way ANOVA with Bonferroni correction (“multiple comparisons, corrected with Bonferroni test”) and visualized in GraphPad Prism v. 8 (https://www.graphpad.com).

**Table 1.**
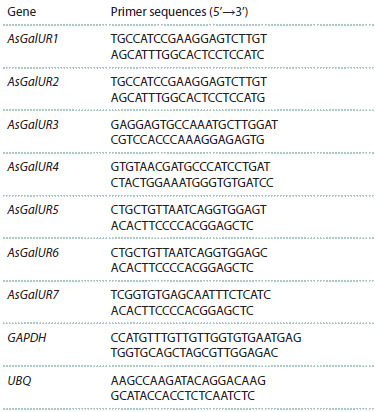
Primer sequences for qRT-PCR

Analysis of the AsGalUR gene expression dynamics in garlic seedlings exposed to various stressors (drought, salinity, cold) and exogenous phytohormones. Garlic cv. Scorpion plants were grown in transparent glass beakers in water until 15-day-old seedlings were obtained; cloves were fixed in a porous polyethylene substrate so that only the lower part of the clove (the root zone) was submerged in water. At 9:00 and 15:00 of the light growth phase, leaf and root samples were collected (stored at –80 °C) for subsequent analysis (qRT-PCR) of AsGalUR gene expression.

To imitate stressful conditions, experimental plants were transferred to the corresponding aqueous solutions for 24 h (100 mM NaCl for salinity; 10 % PEG-6000 for drought; 100 μM abscisic acid (ABA) and 100 μM methyl jasmonate (MeJA) for exogenous phytohotmones). Control plants remained in water. For cold exposure, experimental plants were placed in a climate chamber (+4 °C, without light), while the control plants were maintained in the dark at 22 °C. After 6 h and 24 h of exposure to the stressor/hormone, roots and leaves were collected from plants in the experimental and control groups and stored at –80 °C.

The collected plant material was ground in liquid nitrogen and used to obtain RNA/cDNA preparations and perform qRT-PCR as described above.

Determination of ascorbate content in plant tissues. The AsA content (mg/g fresh weight) was measured in the roots and leaves of garlic plants subjected to stress and treatment with phytohormones. Analysis was performed using the L-Ascorbic acid kit (R-Biopharm AG, Germany), and absorption spectra were recorded on an Eppendorf BioSpectrometer® basic spectrophotometer (Eppendorf, Germany). Regression analysis of the data (search for correlations between the expression level of AsGalUR genes and ascorbate content) was performed using GraphPad Prism v. 8 (https://www.graphpad.com).

## Results

**Identification and structural characterization of garlic D-galacturonate reductase genes**

Twenty-seven genes encoding AKR family proteins were identified in the A. sativum genome, seven of which were homologous to GalUR in S. lycopersicum (LOC101256763 and LOC101250974) and F. × ananassa (AF039182.1) and annotated in AlliumDB as “KEGG pathway: D-galacturonate reductase”. These seven garlic genes were numbered AsGalUR1– 7 in the order of their localization on chromosomes 5, 6, and 7. The genes differed in the number of exons – 3 (AsGalUR5–7) or 4 (AsGalUR1–4), and in size – from 1,105 bp (AsGalUR5) to 4,085 bp (AsGalUR7). The genes were similar in terms of the length of the coding sequence (CDS) and the predicted physicochemical properties of the encoded proteins (Table 2).

**Table 2.**
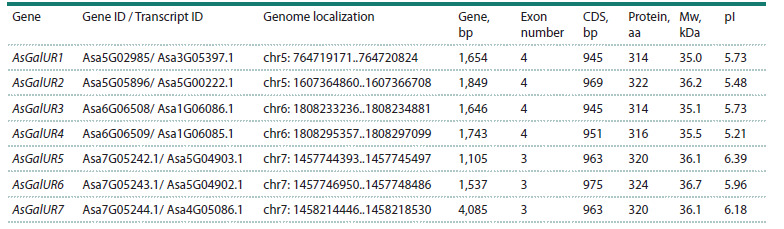
Characteristics of AsGalUR genes

**Fig. 1.**
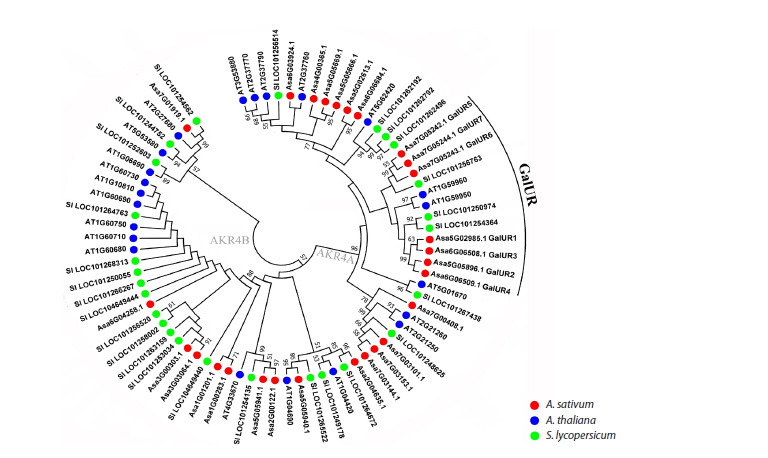
Dendrogram constructed based on the amino acid sequences of AKR family proteins from garlic (A. sativum), Thale cress (A. thaliana), and tomato (S. lycopersicum) (MEGA 7.0, Neighbor-Joining method, 1,000 bootstrap replicates, significant bootstrap values (%) are indicated at the base of the branches).

To construct a phylogenetic dendrogram, the amino acid sequences of all 27 identified AKRs from A. sativum were compared with AKR homologs from S. lycopersicum and A. thaliana. In the resulting dendrogram, the proteins were divided into two subfamilies, AKR4A and AKR4B (Fig. 1). The AsGalUR1–7 sequences formed a separate clade within AKR4A, dividing into subclades I (AsGalUR1–4) and II (AsGalUR5–7). The first (I) turned out to be homologs of D-galacturonate reductases from A. thaliana (AT1G59950 and AT1G59960 (Duan et al., 2020)) and S. lycopersicum SlAKR4B (LOC101250974 (Suekawa et al., 2016)), as well as of an uncharacterized S. lycopersicum AKR (LOC101254364). The second (II) clustered with known S. lycopersicum GalUR (LOC101256763 (Suekawa et al., 2016)) (Fig. 1). The homology of AsGalUR sequences was 86–99 % (I) and 89–95 % (II) within subclades, and 48–52 % between subclades I and II.

**Fig. 2.**
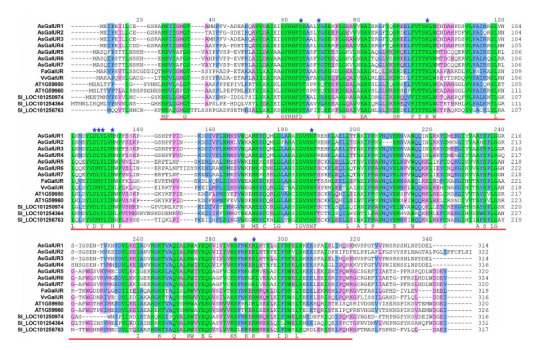
Alignment of the amino acid sequences of D-galacturonate reductases from A. sativum (As), F. × ananassa (Fa), V. vinifera (Vv), A. thaliana (AT), and S. lycopersicum (Sl). The background color corresponds to the level of amino acid conservation in the analyzed proteins (green – 100 %, blue – 80 %, pink – 60 %). The position of the AKR domain PF00248.18 is indicated by red underlining. Blue asterisks indicate active sites and cofactor-binding sites of plant AKRs (according to (Suekawa et al., 2016)).

All the AsGalUR1–7 proteins were found to contain the conserved AKR domain PF00248.18 (the domain position was determined by comparison with homologs from S. lycopersicum, Vitis vinifera L., F. × ananassa, and A. thaliana) and, according to (Suekawa et al. 2016), 10 functionally important amino acid residues (aa), including the plant AKR cofactor binding sites (Fig. 2).

**Fig. 3.**
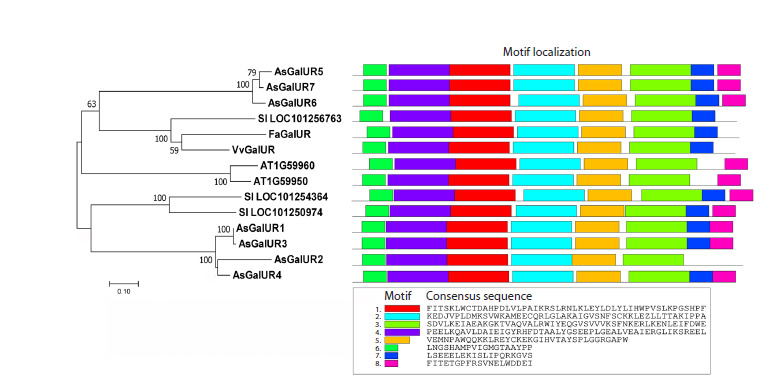
Phylogenetic relationships and comparative profiling of conserved motifs of D-galacturonate reductases from A. sativum (As), F. × ananassa (Fa), V. vinifera (Vv), A. thaliana (AT) и S. lycopersicum (Sl).

In the same proteins, conserved motifs were also identified (Fig. 3). The composition and position of the motifs were similar within the group of analyzed proteins, with the exception of slightly different C-terminal consensuses, which is associated with variability in the extra-domain region. Namely, individual proteins lacked motif 7 (AT1G59950 and AT1G59960) or 8 (Sl_LOC101256763, FaGalUR, and VvGalUR), or both motifs (AsGalUR2).

Analysis of AsGalUR1–7 sequences in PANNZER predicted that all seven proteins possess oxidoreductase activity (GO:0016616) and are localized in the cytosol (GO:0005829).

**Determination of the AsGalUR1–7 gene expression pattern in garlic plants**

**Fig. 4.**
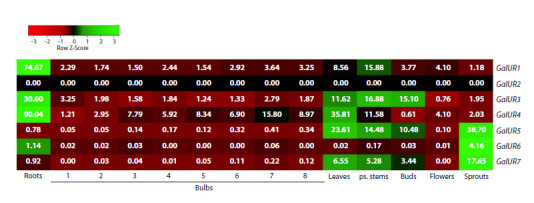
Heatmap of AsGalUR1–7 expression in different organs of garlic A. sativum cv. Ershuizao based on transcriptome data (Sun et al., 2020). Numbers in boxes indicate the average FPKM values for three biological replicates. For bulbs, stages 1–8 (corresponding to 192, 197, 202, 207, 212, 217, 222, and 227 days of development) are shown

Using A. sativum cv. Ershuizao transcriptome data (Sun et al., 2020), the expression profiles of the AsGalUR1–7 genes were determined in various garlic organs (including stages 1–8 of bulb growth and development) (Fig. 4). It was found that the AsGalUR1, 3, and 4 genes (subclade I) were expressed in all analyzed organs, with the highest levels in roots (all three genes), leaves (AsGalUR3 and 4), stems (AsGalUR1 and 3), and buds (AsGalUR3). Transcripts of the fourth gene of subclade I, AsGalUR2, were not detected in any of the organs analyzed.

Transcripts of the AsGalUR5–7 genes (subclade II) were present predominantly in aboveground organs (except flowers), with a maximum in seedlings (all three genes). AsGalUR5 and 7 (but not AsGalUR6) were also highly expressed in leaves stems, and buds. In bulbs and roots, the expression level of the AsGalUR5–7 genes was low relative to the aboveground parts of the plant (Fig. 4).

Overall, based on the preferred expression profile (Fig. 4), the genes were conditionally assigned to the root (AsGalUR1, 3, and 4, despite a significant number of transcripts also being expressed in leaves/stems) and leaf (AsGalUR5–7) groups, which corresponded to their distribution among phylogenetic subclades I and II (Fig. 1). The exception was the AsGalUR2 gene of subclade I (Fig. 1), for which no expression was detected in garlic organs (Fig. 4).

**Fig. 5.**
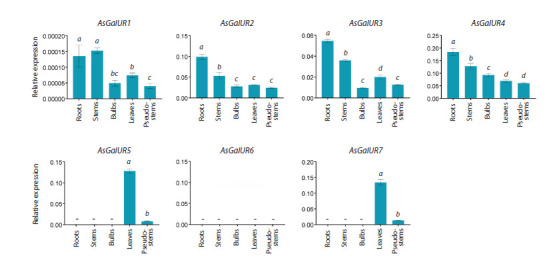
Expression profile (qRT-PCR) of the AsGalUR1–7 genes in cv. Scorpion garlic plants. Significant differences in gene expression levels between different organs at a–dp <0.05.

In the organs of garlic cv. Scorpion plants, AsGalUR1–7 gene expression was determined using qRT-PCR (Fig. 5). In contrast to the transcriptome data (Fig. 4), transcripts of the AsGalUR2 gene were detected everywhere, while AsGalUR6 was not expressed. Subclade I genes (AsGalUR1–4) were expressed in all analyzed organs, with a maximum in the roots and clove base. Transcripts of subclade II genes (AsGalUR5 and 7) were detected only in the leaves (maximum) and pseudostem (Fig. 5).

Thus, the results of qRT-PCR (Fig. 5) made adjustments to the in silico expression profile of the AsGalUR2 and 6 genes (Fig. 4) and confirmed the possibility of dividing the genes of subclades I and II into genes with predominant expression in the underground (I) and aboveground (II) parts of the plant.

**Determination of the expression pattern of the AsGalUR1–7 genes in garlic seedlings: 6-h dynamics during the light phase of plant growth and response to stress factors and exogenous phytohormones**

**Fig. 6.**
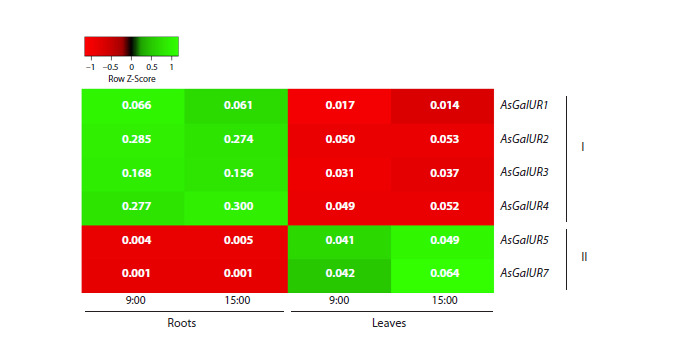
Heatmap of the expression (based on qRT-PCR results) of the AsGalUR1–5 and 7 genes in the roots and leaves of 15-day-old garlic cv. Scorpion seedlings at two time points of the plant growth light phase – 9:00 and 15:00. The numbers in boxes indicate the average values of the AsGalUR gene expression levels, normalized to the expression of the reference genes GAPDH and UBQ.

The AsGalUR1-5 and 7 gene expression levels were determined in roots and leaves of 15-day-old garlic cv. Scorpion seedlings at two time points (9:00 and 15:00) of the light growth phase (Fig. 6). It was confirmed that the genes of root subclade I (AsGalUR1-4) are most active in roots, while the genes of leaf subclade II (AsGalUR5 and 7) are most active in leaves. Moreover, over the 6-h light phase of plant growth, the gene expression level changed insignificantly, maintaining organ specificity. Interestingly, the transcripts of the “leaf” genes AsGalUR5 and 7, which in adult plants were present only in aboveground organs (Fig. 5), were also detected in the roots of seedlings, although with a significantly lower (20–25 times) expression level than in the leaves (Fig. 6).

**Fig. 7.**
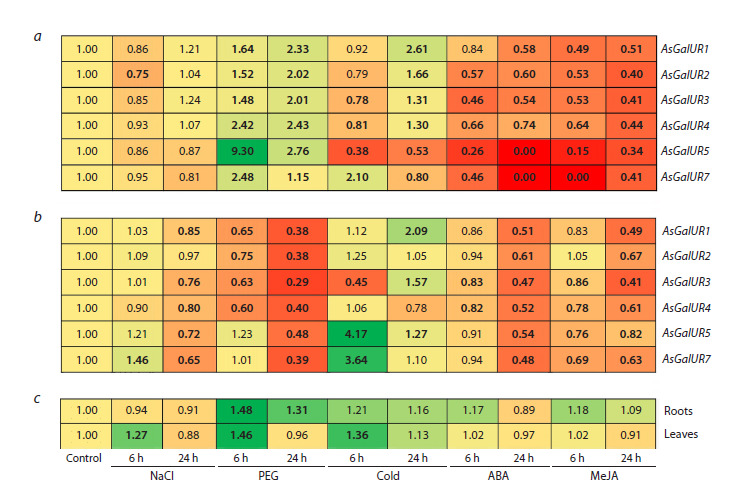
Dynamics of AsGalUR gene expression in roots (a) and leaves (b) of garlic seedlings in response to abiotic stressors (salinity, 100 mM NaCl; drought, 10 % PEG-6000; cold stress, +4 °C) and exogenous phytohormones (100 μM ABA; 100 μM MeJa). Changes in the AsA content (c) in the roots and leaves of the same garlic seedlings in response to abiotic stressors and phytohormones. Values significantly ( p <0.05) different from the control are shown in bold. Gradient coloring of cells corresponds to changes in gene expression level towards a decrease (shades of red) or an increase (shades of green) in relation to the control (yellow).

To determine the involvement of D-galacturonate reductases in the stress response of garlic plants, 15-day-old cv. Scorpion seedlings were exposed to various stressors (salinity, drought, cold, and the exogenous phytohormones ABA and MeJA). After 6 h and 24 h of exposure, gene expression of AsGalUR1–5 and 7 was analyzed in the roots and leaves (Fig. 7).

In roots, it was shown that excess NaCl either did not change (AsGalUR1, 3–5, and 7) or suppressed (AsGalUR2, 6 h under stress conditions) gene expression. Dehydration (PEG) significantly stimulated gene expression (by 1.5–9.3 times) at both time points, with the exception of AsGalUR7, the transcript levels of which approached control values after 24 h. Six hours of cold exposure affected individual genes, decreasing (AsGalUR3, 4, and 5) or increasing (AsGalUR7) their expression. However, after 24 h, the expression of all genes changed in a subclade-specific manner: it increased (AsGalUR1–4, subclade I) or decreased (AsGalUR5 and 7, subclade II) compared to the control level. Phytohormone treatment significantly reduced the expression of all genes, reaching zero for AsGalUR5 and 7 (Fig. 7a).

In leaves, 6 h of exposure to salt stress stimulated the expression of AsGalUR7 (by 1.5 times), while 24 h of exposure suppressed the activity of all genes (except AsGalUR2). Six hours of drought did not change (AsGalUR5 and 7) or led to a decrease (AsGalUR1–4) in gene expression; after 24 h, the transcript levels of AsGalUR1–5 and 7 decreased by 2.5–3.4 times. In response to 6 h of cold conditions, gene expression increased (AsGalUR5 and 7), decreased (AsGalUR3), or did not change (AsGalUR1, 2, and 4); 24 h of exposure led to an increase in the expression of AsGalUR1, 3, and 5. Treatment with ABA and MeJA, as in roots, negatively affected the expression of the AsGalUR1–5 and 7 genes (Fig. 7b).

In garlic seedlings exposed to stress factors and exogenous phytohormones, the ascorbate content was determined to be 1.4–2.2 and 12.1–24.3 mg/100 g in roots and leaves, respectively (Fig. 7c). In roots, AsA content increased at both time points (6 h and 24 h of stress) in response to drought (PEG), whereas it did not change under the influence of other stressors and phytohormones. In leaves, increased ascorbate accumulation was observed after 6 h of exposure to NaCl, drought, and cold stress; at 24 h, the AsA content returned to control values (Fig. 7c).

In the leaves of garlic seedlings, the correlation analysis revealed a significant ( p < 0.05) dependence of the ascorbate content on the expression level of the AsGalUR1 (correlation coefficient r = 0.63) and AsGalUR4 (r = 0.66) genes under stress conditions

## Discussion

The D-galacturonic pathway for L-ascorbic acid biosynthesis in plants was first discovered in the 1950s, and the enzyme D-galacturonate reductase was discovered at the same time. It catalyzes the reduction of D-galacturonic acid methyl ester to L-galactonic acid (Isherwood, Mapson, 1956), which is converted in several stages to AsA (Ishikawa et al., 2008; Peltonen, Richard, 2022). In 2003, the first gene of the GalUR family encoding D-galacturonate reductase was cloned in strawberry F. × ananassa (FaGalUR), in which ripe berries ascorbate is synthesized predominantly via the D-galacturonic pathway (Agius et al., 2003; Cruz-Rus et al., 2011).

In this study, the AKR4 gene family (Fig. 1) was identified for the first time in the genome of garlic A. sativum. Seventeen of the 27 identified AsAKR genes belonged to the AKR4A subfamily, which was consistent with the higher number of AKR4A genes compared to AKR4B in higher plants (Duan et al., 2020). Seven AKR4A genes, AsGalUR1–7 (Fig. 1, Table 2), encoded homologs of known GalUR proteins from S. lycopersicum (Suekawa et al., 2016), A. thaliana (Duan et al., 2020), and F. × ananassa (Agius et al., 2003).

Based on structural homology, it was suggested that AsGalUR1– 7 proteins may function as D-galacturonate reductases in garlic plants. The presence of functionally important sites and a conserved domain specific for AKR in all AsGalUR1-7 proteins (Fig. 2), along with the results of GO analysis, also indicates the presence of oxidoreductase activity characteristic of AKR in AsGalUR1–7 when localized in the cell cytosol. The predicted localization of AsGalUR1–7 was consistent with the fact that the two-step conversion of D-galacturonate to L-galactono-1,4-lactone involving GalUR occurs precisely in the cytosol (Smirnoff et al., 2001).

Based on the number of exons, the genes were divided into two groups: AsGalUR1–4 (4 exons) and AsGalUR5–7 (3 exons) (Table 2), which suggested the existence of functional differences between them, possibly associated with the specificity of expression for individual organs/tissues/ developmental stages and/or with the adaptive reactions of the garlic plant. This division was confirmed by phylogenetic analysis. In the dendrogram, the AsGalUR1–7 proteins formed two subclades: I (AsGalUR1-4) and II (AsGalUR5-7) (Fig. 1), although the set of conserved motifs looked identical, with the exception of the C-terminal part of AsGalUR2 (Fig. 3). The variability of the AsGalUR2 C-terminal sequence may indicate the presence of individual functional properties in this protein, but does not exclude its involvement in the synthesis of AsA. The different numbers of subclade I and II GalUR genes in A. sativum and, for example, S. lycopersicum, A. thaliana or Brassica rapa L. (Duan et al., 2020) (Fig. 1) suggests the emergence of precursors of these genes before the separation of the monocot and dicot classes and subsequent species-specific duplication evolution of the corresponding gene families.

As mentioned above, activation of D-galacturonic acid pathway of AsA synthesis as an alternative to the main L-galactose pathway occurs in ripe, juicy fruits, which is associated with the degradation of cell wall pectin in the pulp with the release of D-galacturonate (a substrate for GalUR) (Cruz-Rus et al., 2011; Badejo et al., 2012). Growing and ripening fruits obtain the required amount of ascorbate through internal synthesis via the main pathway and translocation of AsA from the leaves (Badejo et al., 2012). Softening of ripe fruits is accompanied by the destruction of cell walls. During this, pectin, which makes up ∼35 % of the primary walls in dicotyledons and non-cereal monocots (including garlic) (Mohnen, 2008) and consists of ∼70 % galacturonic acid residues (Mølhøj et al., 2004; Cruz-Rus et al., 2011; Badejo et al., 2012), is degraded. There is a significant increase in the amount of substrate for GalUR, which stimulates a switch to an alternative pathway for AsA synthesis in fruits (Peltonen, Richard, 2022).

Thus, to activate the D-galacturonic acid pathway, plants require D-galacturonate as a substrate for GalUR (Peltonen, Richard, 2022). However, in vegetative tissues, as well as in growing storage organs, this compound is strictly necessary for the synthesis of cell wall pectin, since plant growth and development are accompanied by active cell division (Mohnen, 2008). Nevertheless, the constant presence of high concentrations of D-galacturonate in the cytosol of vegetative tissue cells suggests that D-galacturonic acid pathway of AsA synthesis may also occur there as a minor addition to the main L-galactose pathway. This is indirectly confirmed by the positive correlation between the content of AsA and the expression level of D-galacturonate reductase genes found in the leaves of B. rapa (Duan et al., 2020) and tea bush (Camellia sinensis (L.) O. Kuntze) (Li et al., 2017).

Based on the above, in this study, we analyzed the expression of the AsGalUR1–7 genes in various organs of the A. sativum cv. Ershuizao plant (in silico, using transcriptome data (Sun et al., 2020)) (Fig. 4), as well as in the leaves and roots (qRT-PCR) of the garlic cv. Scorpion, including in response to abiotic stressors and exogenous phytohormones (Fig. 5–7). In addition to vegetative organs, bulb samples (as a storage organ) at several stages of growth and maturation were included in the in silico analysis (Sun et al., 2020).

The analysis confirmed that the groups of AsGalUR genes belonging to subclades I and II differ in their expression profiles and, presumably, in their functional specialization. So, the transcript levels of genes AsGalUR1–4 (I) were significantly higher in the underground part of the garlic plant compared to the aboveground organs, while the opposite pattern was observed for genes AsGalUR5–7 (II) (Figs. 4–6). This suggests organ-specific involvement of these genes in the D-galacturonic pathway; subclade I and II genes were provisionally named as root (AsGalUR1–4) and leaf (AsGalUR5–7) genes. A question remains regarding the AsGalUR2 and 6 genes, which showed different expression patterns depending on the type of analysis, but based on the totality of the data (Fig. 4 and 5), these genes correspond to the characteristics of their groups, although they require further study. In our further work, we relied on the results of qRT-PCR, that is, we excluded AsGalUR6 from the study.

As expected, in the bulb, the root genes AsGalUR1–4 were expressed significantly higher than the leaf genes; however, no significant trends in transcript level changes were observed as the bulbs approached maturity (Fig. 4). This may be due to differences in the definition of full ripeness between juicy fruits and root vegetables. While in fruits biological ripeness is accompanied by softening of the pulp (Badejo et al., 2012), in root vegetables it means enlargement and vacuolization of cells for nutrient accumulation and the onset of physiological dormancy (Teper-Bamnolker et al., 2012). Softening of root tissue may be a sign of dormancy release, wilting, or rotting

Consistent with the fact that 12 GalUR genes of B. rapa exhibit obvious divergence of expression under different stress conditions (Duan et al., 2020), in our study, the AsGalUR1–5 and 7 genes also responded diversely and organ-dependently to both abiotic stressors and exogenous ABA and MeJA (Fig. 7a, b). Despite the conditional division of genes into root and leaf groups, in most cases their expression in response to stress changed in both roots and leaves (Fig. 7a, b). This indicates the involvement of all AsGalUR1–5 and 7 genes in AsA synthesis via an alternative pathway under stress conditions throughout the plant, but with predominant specificity in the underground or aboveground parts

Exogenous phytohormones suppressed the expression of genes of both group in both tissue types (Fig. 7a, b), which is consistent with the role of ABA and MeJA as plant growth regulators and cell division stimulators (Fattorini et al., 2009; Xie et al., 2020). It is possible that hormone treatment accelerates cell division, which requires increased pectin synthesis, reduces the amount of substrate for GalUR and, as a result, downregulates the expression of AsGalUR genes. On the other hand, exogenous ABA has been shown to stimulate AsA synthesis (Xu et al., 2022); however, this most likely relates to the main ascorbate synthesis pathway. In addition, we did not observe an increase in ascorbate content in response to phytohormones (Fig. 7c), which may suggest the absence of an effect or a delayed stimulatory effect of ABA on AsA synthesis.

The effects of abiotic stressors on gene expression were more diverse than those of hormones (Fig. 7a, b). The main difference between the reaction of root (AsGalUR1–4) and leaf (AsGalUR5, 7) genes was the opposite expression dynamics under low temperature exposure. In roots (as expected from conditional gene specialization), after 24 h of treatment, the expression of root genes increased, while that of leaf genes decreased (Fig. 7a). In leaves, on the contrary, after 6 h of exposure, the expression of leaf AsGalUR5 and 7 sharply increased, while the expression of root AsGalUR1–4 decreased or remained unchanged (Fig. 7b). This is consistent with the positive dependence of plant cold resistance on AsA content (Fu et al., 2023) and emphasizes the organ specificity of the increase in AsGalUR gene expression in response to cold, which led to a significant (leaves) or weaker (roots) increase in ascorbate content (Fig. 7c).

Ascorbic acid promotes plant tolerance to salinity and drought (Younis et al., 2010). Accordingly, the expression of AsGalUR genes changed in response to osmotic/ionic stress (PEG, NaCl) with a more pronounced effect in the roots, which is associated with the specificity of stress conditions created (roots immersed in a PEG or NaCl solution). However, no functional differences were found between leaf and root AsGalUR genes: in leaves, the expression level of all genes decreased, while in roots it did not change (NaCl) or increased (PEG) (Fig. 7a, b), which was accompanied by an increase in AsA accumulation in organs of both types (Fig. 7c). It should be noted that the correlation between the gene expression level and the ascorbate content under stress conditions was found only in leaves and only for the AsGalUR1 and 4 genes assigned to the root group, which confirms the conventionality of the functional division of AsGalUR genes in the adaptive responses of garlic plants.

The data we obtained are fully consistent with the activation of alternative pathways for ascorbate synthesis in plants under stressful conditions shown in other studies (Xu et al., 2012; Ruggieri et al., 2016). In particular, this may be due to a decrease in the flux of the main L-galactose pathway in response to stressors (due to the active involvement of this pathway precursor, D-glucose, in plant stress responses), which was demonstrated in the analysis of the vtc1 and vtc2 mutants of A. thaliana (Quiñones et al., 2024).

## Conclusion

In this study, we identified the A. sativum AKR gene family and defined seven AsGalUR1–7 genes, which presumably encode D-galacturonate reductases – key enzymes in the alternative D-galacturonic acid pathway of L-ascorbic acid biosynthesis. We characterized the structure and phylogeny of the genes and encoded proteins, as well as the organ-specific expression profile of the AsGalUR1–7 genes. Based on this, the genes were conditionally divided into root (AsGalUR1–4) and leaf (AsGalUR5–7) groups. We analyzed gene expression in response to abiotic stress factors (salinity, drought, and cold) and exogenous phytohormones (ABA, MeJA), which revealed additional functional features of the genes in determining garlic plant stress tolerance.

## Conflict of interest

The authors declare no conflict of interest.

## References

Agius F., González-Lamothe R., Caballero J.L., Muñoz-Blanco J.,
Botella M.A., Valpuesta V. Engineering increased vitamin C levels
in plants by overexpression of a D-galacturonic acid reductase. Nat
Biotechnol. 2003;21(2):177-181. doi 10.1038/nbt777

Anisimova O.K., Shchennikova A.V., Kochieva E.Z., Filyushin
M.A.
Identification and variability of the GDP-L-galactose phosphosphorylase
gene ApGGP1 in leek cultivars. Russ J Genet. 2021a;57(3):
311-318. doi 10.1134/S1022795421030030

Anisimova O.K., Seredin T.M., Shchennikova A.V., Kochieva E.Z.,
Filyushin M.A. Estimation of the vitamin C content and GDP-L-galactose
phosphorylase gene (VTC2) expression level in leek (Allium
porrum L.) cultivars. Russ J Plant Physiol. 2021b;68(1):85-93. doi
10.1134/S1021443720060023

Anisimova O.K., Shchennikova A.V., Kochieva E.Z., Filyushin M.A.
Identification of monodehydroascorbate reductase (MDHAR) genes
in garlic (Allium sativum L.) and their role in the response to Fusarium
proliferatum infection. Russ J Genet. 2022;58(7):773-782. doi
10.1134/S1022795422070031

Badejo A.A., Wada K., Gao Y., Maruta T., Sawa Y., Shigeoka S.,
Ishikawa T. Translocation and the alternative D-galacturonate pathway
contribute to increasing the ascorbate level in ripening tomato
fruits together with the D-mannose/L-galactose pathway. J Exp Bot.
2012;63(1):229-239. doi 10.1093/jxb/err275

Broad R.C., Bonneau J.P., Hellens R.P., Johnson A.A.T. Manipulation
of ascorbate biosynthetic, recycling, and regulatory pathways
for improved abiotic stress tolerance in plants. Int J Mol Sci. 2020;
21(5):1790. doi 10.3390/ijms21051790

Cruz-Rus E., Amaya I., Sánchez-Sevilla J.F., Botella M.A., Valpuesta V.
Regulation of L-ascorbic acid content in strawberry fruits. J Exp Bot.
2011;62(12):4191-4201. doi 10.1093/jxb/err122

Duan W., Huang Z., Li Y., Song X., Sun X., Jin C., Wang Y., Wang J.
Molecular evolutionary and expression pattern analysis of AKR
genes shed new light on GalUR functional characteristics in Brassica
rapa. Int J Mol Sci. 2020;21(17):5987. doi 10.3390/ijms21175987

Fattorini L., Falasca G., Kevers C., Rocca L.M., Zadra C., Altamura
M.M. Adventitious rooting is enhanced by methyl jasmonate in
tobacco thin cell layers. Planta. 2009;231(1):155-168. doi 10.1007/
s00425-009-1035-y

Filyushin M.A., Anisimova O.K., Kochieva E.Z., Shchennikova A.V.
Correlation of ascorbic acid content and the pattern of monodehydroascorbate
reductases (MDHARs) gene expression in leek (Allium
porrum L.). Russ J Plant Physiol. 2021;68(5):849-856. doi 10.1134/
S1021443721050034

Filyushin M.A., Seredin T.M., Shchennikova A.V., Kochieva E.Z. Vitamin
C content and profile of ascorbate metabolism gene expression
in green leaves and bleached parts of the pseudostem of leek
(Allium porrum L.) F1 hybrids. Vavilovskii Zhurnal Genetiki i Selektsii
= Vavilov J Genet Breed. 2025;29(2):200-209. doi 10.18699/
vjgb-25-23

Franceschi V.R., Tarlyn N.M. L-Ascorbic acid is accumulated in source
leaf phloem and transported to sink tissues in plants. Plant Physiol.
2002;130(2):649-656. doi 10.1104/pp.007062

Fu Q., Cao H., Wang L., Lei L., Di T., Ye Y., Ding C., Li N., Hao X.,
Zeng J., Yang Y., Wang X., Ye M., Huang J. Transcriptome analysis
reveals that ascorbic acid treatment enhances the cold tolerance
of tea plants through cell wall remodeling. Int J Mol Sci. 2023;
24(12):10059. doi 10.3390/ijms241210059

Ha J., Kang Y.G., Lee T., Kim M., Yoon M.Y., Lee E., Yang X., Kim D.,
Kim Y.J., Lee T.R., Kim M.Y., Lee S.H. Comprehensive RNA sequencing
and co-expression network analysis to complete the biosynthetic
pathway of coumestrol, a phytoestrogen. Sci Rep. 2019;
9(1):1934. doi 10.1038/s41598-018-38219-6

Huang J., Wu H., Gao R., Wu L., Wang M., Chu Y., Shi Y., Xiang L.,
Yin Q. Integrated multi-omics analysis reveals glycosylation involving
2-O-β-D-Glucopyranosyl-L-ascorbic acid biosynthesis in Lycium
barbarum. Int J Mol Sci. 2025;26(4):1558. doi 10.3390/ijms
26041558

Isherwood F.A., Mapson L.W. Biological synthesis of ascorbic acid:
the conversion of derivatives of D-galacturonic acid into L-ascorbic
acid by plant extracts. Biochem J. 1956;64(1):13-22. doi 10.1042/
bj0640013

Ishikawa T., Nishikawa H., Gao Y., Sawa Y., Shibata H., Yabuta Y., Maruta
T., Shigeoka S. The pathway via D-galacturonate/L-galactonate
is significant for ascorbate biosynthesis in Euglena gracilis: identification
and functional characterization of aldonolactonase. J Biol
Chem. 2008;283(45):31133-31141. doi 10.1074/jbc.M803930200

Li H., Huang W., Wang G.L., Wang W.L., Cui X., Zhuang J. Transcriptomic
analysis of the biosynthesis, recycling, and distribution
of ascorbic acid during leaf development in tea plant (Camellia
sinensis (L.) O. Kuntze). Sci Rep. 2017;7:46212. doi 10.1038/
srep46212

Liu H., Wei L., Ni Y., Chang L., Dong J., Zhong C., Sun R., Li S.,
Xiong R., Wang G., Sun J., Zhang Y., Gao Y. Genome-wide analysis
of ascorbic acid metabolism related genes in Fragaria × ananassa
and its expression pattern analysis in strawberry fruits. Front Plant
Sci. 2022;13:954505. doi 10.3389/fpls.2022.954505

Mohnen D. Pectin structure and biosynthesis. Curr Opin Plant Biol.
2008;11(3):266-277. doi 10.1016/j.pbi.2008.03.006

Mølhøj M., Verma R., Reiter W.D. The biosynthesis of D-Galacturonate
in plants. functional cloning and characterization of a membraneanchored
UDP-D-Glucuronate 4-epimerase from Arabidopsis. Plant
Physiol. 2004;135(3):1221-1230. doi 10.1104/pp.104.043745

Peltonen K.E., Richard P. Identification of a D-galacturonate reductase
efficiently using NADH as a cofactor. Biotechnol Rep (Amst). 2022;
35:e00744. doi 10.1016/j.btre.2022.e00744

Penning T.M. The aldo-keto reductases (AKRs): overview. Chem Biol
Interact. 2015;234:236-246. doi 10.1016/j.cbi.2014.09.024

Popa P.-M., Băbeanu C., Cosmulescu S.-N. Evaluation of chemical
compounds in local garlic genotypes from southwestern Romania.
Appl Sci. 2024;14(16):6899. doi 10.3390/app14166899

Quiñones C.O., Gesto-Borroto R., Wilson R.V., Hernández-Madrigal
S.V., Lorence A. Alternative pathways leading to ascorbate
biosynthesis in plants: lessons from the last 25 years. J Exp Bot.
2024;75(9):2644-2663. doi 10.1093/jxb/erae120

Richardson A.T., McGhie T.K., Cordiner S.B., Stephens T.T.H., Larsen
D.S., Laing W.A., Perry N.B. 2-O-β-d-Glucopyranosyl l-ascorbic
acid, a stable form of vitamin C, is widespread in crop plants.
J Agric Food Chem. 2021;69(3):966-973. doi 10.1021/acs.jafc.
0c06330

Ruggieri V., Bostan H., Barone A., Frusciante L., Chiusano M.L. Integrated
bioinformatics to decipher the ascorbic acid metabolic network
in tomato. Plant Mol Biol. 2016;91(4-5):397-412. doi 10.1007/
s11103-016-0469-4Sengupta D., Naik D., Reddy A.R. Plant aldo-keto reductases (AKRs)
as multi-tasking soldiers involved in diverse plant metabolic processes
and stress defense: a structure-function update. J Plant Physiol.
2015;179:40-55. doi 10.1016/j.jplph.2015.03.004

Shchennikova A.V., Kochieva E.Z., Filyushin M.A. Ascorbate biosynthesis
and recycling genes are involved in the responses of garlic
Allium sativum L. plants to Fusarium proliferatum infection.
Dokl Biochem Biophys. 2025;520(1):49-52. doi 10.1134/S1607672
924601057

Skoczylas J., Jędrszczyk E., Dziadek K., Dacewicz E., Kopeć A. Basic
chemical composition, antioxidant activity and selected polyphenolic
compounds profile in garlic leaves and bulbs collected at various
stages of development. Molecules. 2023;28(18):6653. doi 10.3390/
molecules28186653

Smirnoff N. Ascorbic acid metabolism and functions: a comparison of
plants and mammals. Free Radic Biol Med. 2018;22:116-129. doi
10.1016/j.freeradbiomed.2018.03.033

Smirnoff N., Wheeler G.L. The ascorbate biosynthesis pathway in
plants is known, but there is a way to go with understanding control
and functions. J Exp Bot. 2024;75(9):2604-2630. doi 10.1093/jxb/
erad505

Smirnoff N., Conklin P.L., Loewus F.A. biosynthesis of ascorbic acid in
plants: a renaissance. Annu Rev Plant Physiol Plant Mol Biol. 2001;
52:437-467. doi 10.1146/annurev.arplant.52.1.437

Šnirc M., Lidiková J., Čeryová N., Pintér E., Ivanišová E., Musilová J.,
Vollmannová A., Rybnikár S. Mineral and phytochemical profiles
of selected garlic (Allium sativum L.) cultivars. South Afr J Bot.
2023;158:319-325. doi 10.1016/j.sajb.2023.05.024

Suekawa M., Fujikawa Y., Inada S., Murano A., Esaka M. Gene expression
and promoter analysis of a novel tomato aldo-keto reductase in
response to environmental stresses. J Plant Physiol. 2016;200:35-44.
doi 10.1016/j.jplph.2016.05.015

Sun X., Zhu S., Li N., Cheng Y., Zhao J., Qiao X., Lu L., … Zhao X.,
Tian S., Su J., Cheng Z., Liu T. A chromosome-level genome assembly
of garlic (Allium sativum) provides insights into genome evolution
and allicin biosynthesis. Mol Plant. 2020;13(9):1328-1339. doi
10.1016/j.molp.2020.07.019

Teper-Bamnolker P., Buskila Y., Lopesco Y., Ben-Dor S., Saad I., Holdengreber
V., Belausov E., Zemach H., Ori N., Lers A., Eshel D.
Release of apical dominance in potato tuber is accompanied by programmed cell death in the apical bud meristem. Plant Physiol. 2012;
158(4):2053-2067. doi 10.1104/pp.112.194076

Vargas J.A., Leonardo D.A., D’Muniz Pereira H., Lopes A.R., Rodriguez
H.N., Cobos M., Marapara J.L., Castro J.C., Garratt R.C. Structural
characterization of L-galactose dehydrogenase: an essential
enzyme for vitamin C biosynthesis. Plant Cell Physiol. 2022;63(8):
1140-1155. doi 10.1093/pcp/pcac090

Xie Q., Essemine J., Pang X., Chen H., Cai W. Exogenous application
of abscisic acid to shoots promotes primary root cell division
and elongation. Plant Sci. 2020;292:110385. doi 10.1016/j.plantsci.
2019.110385

Xu X., Zhang Q., Gao X., Wu G., Wu M., Yuan Y., Zheng X., … Qi T.,
Li H., Luo Z., Li Z., Deng W. Auxin and abscisic acid antagonistically
regulate ascorbic acid production via the SlMAPK8-SlARF4-
SlMYB11 module in tomato. Plant Cell. 2022;34(11):4409-4427.
doi 10.1093/plcell/koac262

Yenealem D., Eyayu D., Tibebe D., Mulugeta M., Kassa Y., Moges Z.,
Kerebih F., Fentie T., Amare A., Ayalew M. Electrochemical characterization
and detection of ascorbic acid in garlic using activated
glassy carbon electrode: a comprehensive study. Food Anal Methods.
2024;17:1473-1483. doi 10.1007/s12161-024-02660-3

Younis M.E., Hasaneen M.N., Kazamel A.M. Exogenously applied
ascorbic acid ameliorates detrimental effects of NaCl and mannitol
stress in Vicia faba seedlings. Protoplasma. 2010;239(1-4):39-48.
doi 10.1007/s00709-009-0080-5

